# Supplementary material for: Experimental evidence of tetrahedral symmetry breaking in SiO2 glass under pressure
Source: Nat Commun. 2022 Apr 28;13:2292. doi: 10.1038/s41467-022-30028-w (PMC9051114; doi:10.1038/s41467-022-30028-w)
Supplement: Supplementary file 1 — Supplementary Information [file 41467_2022_30028_MOESM1_ESM.pdf]

Supplementary Information

Experimental evidence of tetrahedral symmetry breaking in SiO_2 glass under pressure

Kono et al.

Supplementary figures

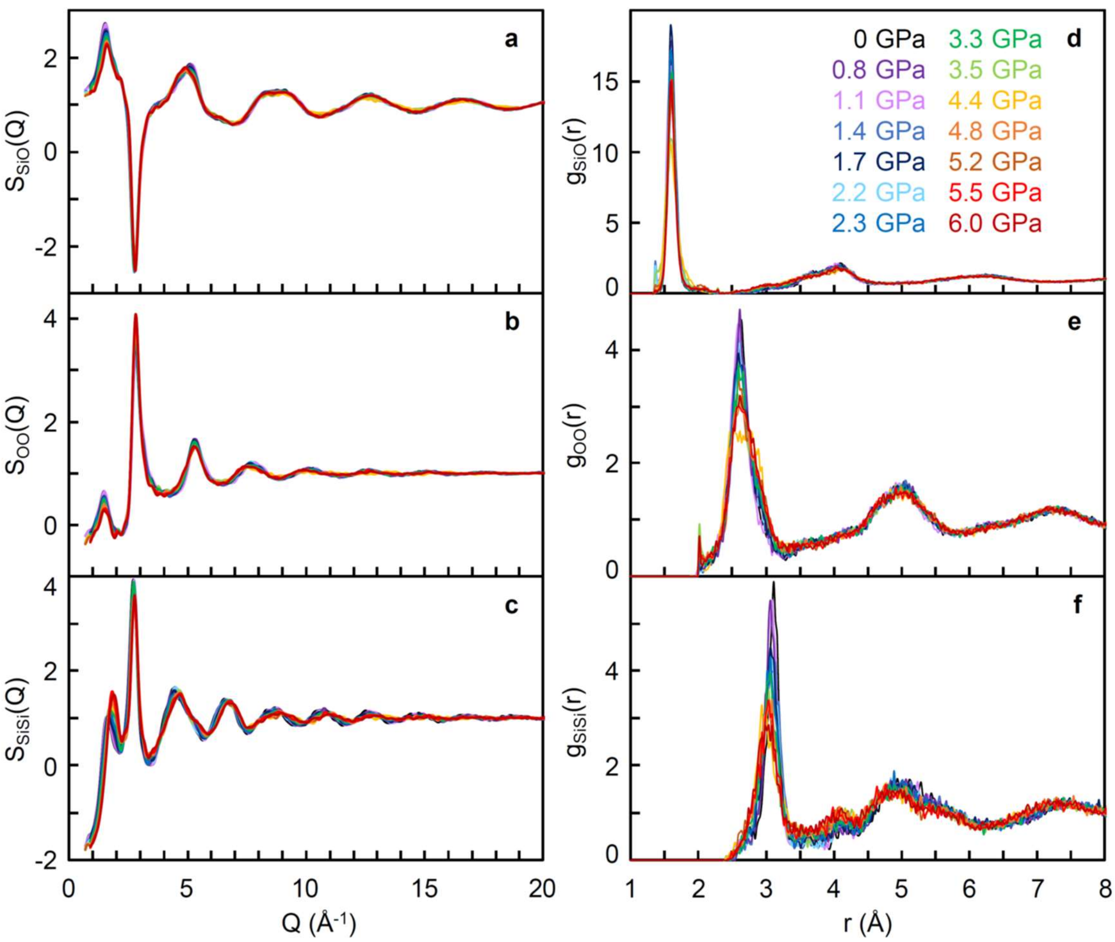

Supplementary figure 1. Partial structure factors and partial pair distribution functions obtained by the MD-RMC modelling on the experimentally observed structure factors [S(Q)] of SiO_2 glass under pressure. Partial structure factors and partial pair distribution functions of Si-O [$S_{SiO}(Q)$ (**a**) and $g_{SiO}(r)$ (**d**)], O-O [$S_{OO}(Q)$ (**b**) and $g_{OO}(r)$ (**e**)], and Si-Si ($S_{SiSi}(Q)$ (**c**) and $g_{SiSi}(r)$ (**f**)], respectively.

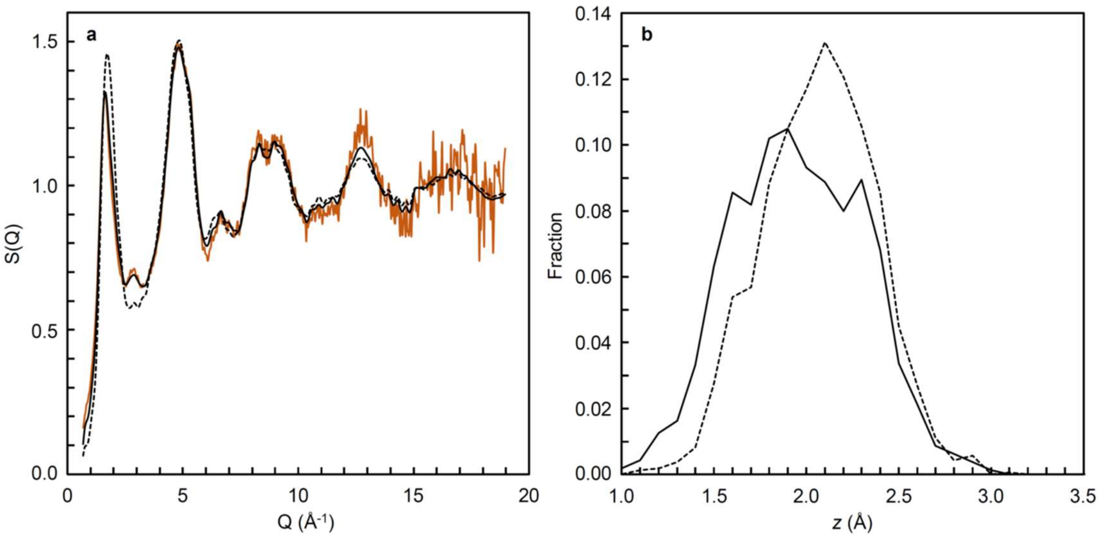

Supplementary figure 2. A simulated result of the influence of the second peak of structure factor [S(Q)] at ~2.9 Å^{-1} on the distribution of the parameter *z* in SiO_2 glass. **a** Orange line shows experimentally observed S(Q) of SiO_2 glass at 5.2 GPa, and two black lines represent S(Q) of two MD-RMC models. Solid black line represents S(Q) of the final MD-RMC model, which reproduces the experimentally observed S(Q) well including the second peak at ~2.9 Å^{-1}. On the other hand, a simulated MD-RMC model shown by broken black line reproduces most of the oscillation features of the experimentally observed S(Q), while it does not reproduce the second peak at ~2.9 Å^{-1}. The difference in the second peak of S(Q) at ~2.9 Å^{-1} between two MD-RMC models yields marked difference in the distribution of the parameter *z* at 1.6-1.7 Å (**b**).

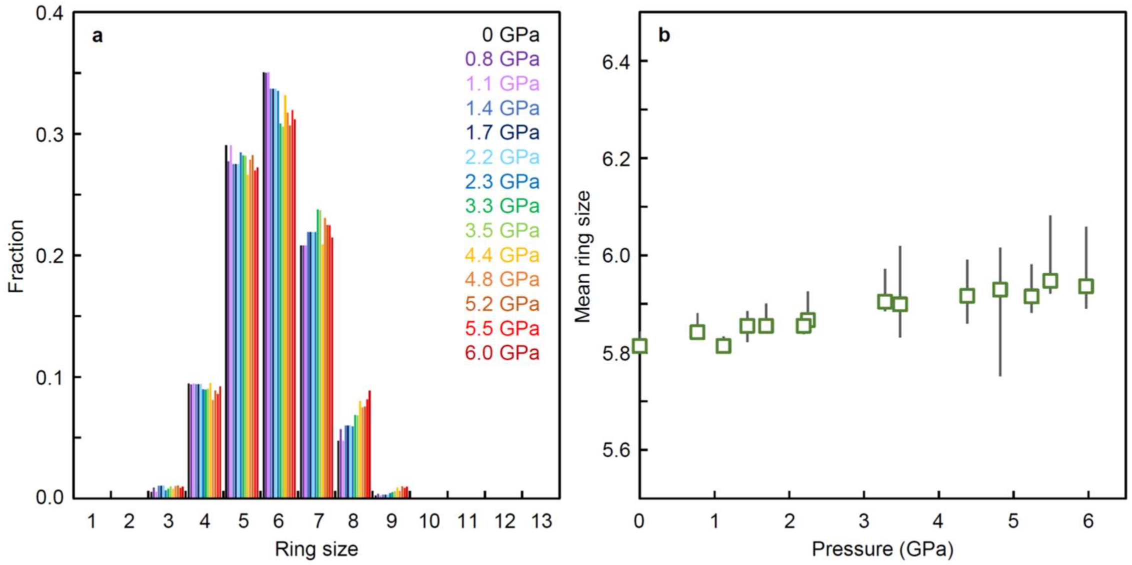

Supplementary figure 3. Ring size statistics analysis in SiO_2 glass under pressure. a Distributions of ring sizes in SiO_2 glass under pressure, calculated using the shortest-path analysis[1]. This analysis counts the number of atoms in a loop from an atom to the same atom through the shortest-path length without counting a larger number of rings that can be divided into smaller ring size. Multiple counting was avoided by checking the linkage in individual rings. We define an n-membered ring as a ring consisting of n polyhedral and counted up to 13-membered rings. The maximum coordination distance for Si-O correlation is defined as 2.3 Å. We tested influence of the maximum coordination distance for Si-O correlation between 2.2 and 2.4 Å on the ring size distribution, and we observed no marked change in the ring size distribution. The largest fraction of ring size in SiO_2 glass is 6-membered ring, and there is no marked change in the ring size distributions with varying pressure. **b** Mean ring size in SiO_2 glass as a function of pressure. The vertical bars represent error of the mean ring size, which is estimated from the variation of the ring size distribution with varying the maximum coordination distance for Si-O correlation between 2.2 and 2.4 Å. The mean ring size shows subtle change within the errors with increasing pressure. Our observation is consistent with the result of MD simulations[2], who also reported subtle change in the mean ring size with increasing pressure below 10 GPa.

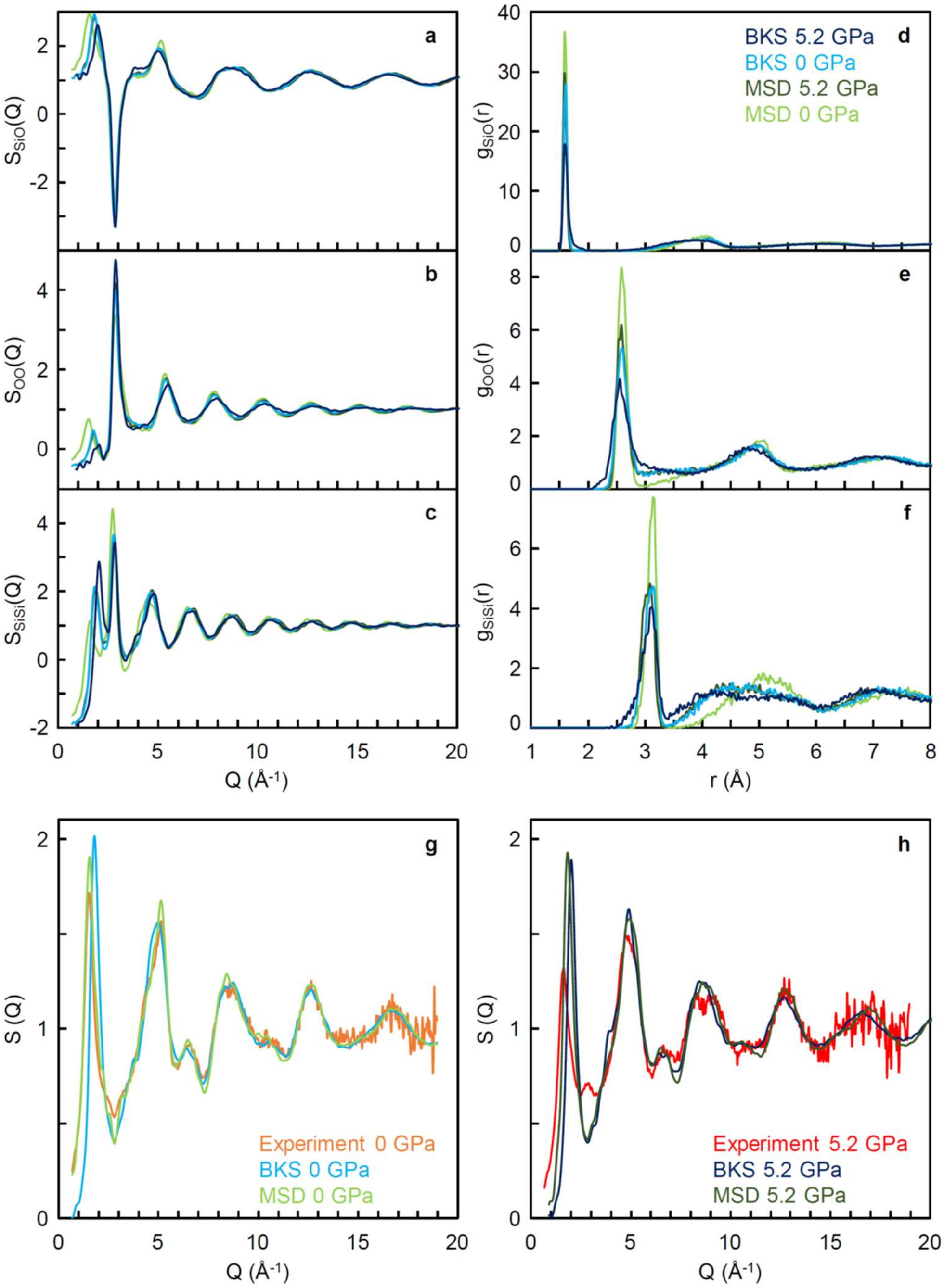

Supplementary figure 4. Partial structure factors and partial pair distribution functions of SiO_2 glass obtained by MD simulations using the BKS and MSD models at 0 and 5.2 GPa. Partial structure factors and partial pair distribution functions of Si-O [$S_{SiO}(Q)$ (**a**) and $g_{SiO}(r)$ (**d**)], O-O [$S_{OO}(Q)$ (**b**) and $g_{OO}(r)$ (**e**)], and Si-Si ($S_{SiSi}(Q)$ (**c**) and $g_{SiSi}(r)$ (**f**)], respectively. **g, h** Comparisons of structure factors [S(Q)] between experiment and MD simulations at 0 GPa (**g**) and 5.2 GPa (**h**).

Supplementary References

1 Le Roux, S. & Jund, P. Ring statistics analysis of topological networks: New approach and application to amorphous GeS_2 and SiO_2 systems. *Computational Materials Science* **49**, 70-83 (2010).

2 Zeidler, A. *et al.* High-pressure transformation of SiO_2 glass from a tetrahedral to an octahedral network: A joint approach using neutron diffraction and molecular dynamics. *Physical review letters* **113**, 135501 (2014).
